# Genome Sequence of *Bacillus thuringiensis* subsp. *kurstaki* Strain HD-1

**DOI:** 10.1128/genomeA.00613-14

**Published:** 2014-07-17

**Authors:** Michael Day, Mohamed Ibrahim, David Dyer, Lee Bulla

**Affiliations:** a Department of Microbiology and Immunology, Oklahoma University Health Sciences Center, Oklahoma City, Oklahoma, USA; b Department of Molecular and Cell Biology, University of Texas at Dallas, Richardson, Texas, USA

## Abstract

We report here the complete genome sequence of *Bacillus thuringiensis* subsp. *kurstaki* strain HD-1, which serves as the primary U.S. reference standard for all commercial insecticidal formulations of *B. thuringiensis* manufactured around the world.

## GENOME ANNOUNCEMENT

*Bacillus thuringiensis* is a ubiquitous Gram-positive bacterium that has been isolated from a variety of ecological niches. The most distinctive property of *B. thuringiensis* is its production of parasporal insecticidal proteins (Cry toxins) whose entomopathogenicity spans a number of insect species among the orders *Lepidoptera* (moths and butterflies), *Diptera* (mosquitoes and blackflies), *Coleoptera* (beetles), and *Hymenoptera* (wasps, ants, and others) (1). These features of *B. thuringiensis* have been exploited for more than 50 years to control a number of insects, including agriculturally and medically important pest and disease vector insects. Indeed, *B. thuringiensis* exhibits highly specific and selective entomopathogenicity and is safe for humans (2–5). Among all strains of *B. thuringiensis*, the HD-1 strain (serotypes 3a and 3b) has been the most intensely used environmentally compatible biopesticide worldwide, and it has been designated the primary U.S. reference standard for the standardization of all commercial formulations of *B. thuringiensis* produced globally (6).

Total genomic DNA was extracted using the high-salt SDS method (7), followed by purification using the phenol-chloroform-isoamyl alcohol protocol (24:24:1 [vol/vol/vol]), as described previously (8). Sequencing was performed on an Illumina MiSeq, using a 300-bp paired-end library, by the Oklahoma Medical Research Foundation Core Facility (http://omrf.org/research-faculty/core-facilities/), generating 39,682,366 reads. Read quality was assessed with FastQC (http://www.bioinformatics.babraham.ac.uk/projects/fastqc/). The MiSeq reads were *de novo* assembled using the A5 assembly pipeline (9), yielding 194 contigs, for a total contig length of 6,391,935 bp, with 34.75% G+C content and an *N*_50_ of 96,479 bp. Automated annotation was performed using the RAST server (10). The resulting contig database contains 11 plasmids, including the previously identified pBMB2062 (11), six plasmids highly similar to those found in *B. thuringiensis* subsp. *kurstaki* HD73 (12), two plasmids similar to those found in *B. thuringiensis* subsp. *thuringiensis* 5056 (13), and one plasmid similar to pBMB9741, found in *B. thuringiensis* subsp. *kurstaki* YBT-1520 (14). We also identified a linear bacteriophage very similar to GIL16c (15). Using the *glpF*, *gmk*, *ilvD*, *pta*, *pur*, *pycA*, and *tpi* genes, we confirmed that strain HD1 is in sequence type 8 (ST8) of the *Bacillus cereus* multilocus sequence type database (16). A comparison of strain HD1 with *B. thuringiensis* HD73 indicated that HD1 lacks approximately 500 kb of DNA found in HD73 while containing approximately 450 kb of DNA not found in strain HD73. The remainder of strain HD1 is highly collinear with HD73. In HD-1, there are 25 rRNA genes, 12 of which code for the 16S ribosomal subunit, 4 for the 23S ribosomal subunit, and 9 for the 5S rRNA subunit. The genome has 84 tRNAs, including five pseudo-tRNAs, which may be tRNA remnants that do not function in translation but may be involved in the maintenance of other cellular functions, such as cell wall biosynthesis and antibiotic resistance, among others (17). These data will aid in-depth explorations of the unique phenotypic properties of this important biocontrol agent.

### Nucleotide sequence accession numbers.

This whole-genome shotgun project has been deposited at DDBJ/EMBL/GenBank under the accession no. JMHW00000000. The version described in this paper is version JMHW01000000.
